# Low profile vascular plug for transarterial occlusion of patent ductus arteriosus in small dogs

**DOI:** 10.1111/jvim.15966

**Published:** 2020-11-26

**Authors:** Alma H. Hulsman, Johannes M. P. J. Breur, Viktor Szatmári

**Affiliations:** ^1^ Department of Clinical Sciences Faculty of Veterinary Medicine, Utrecht University Utrecht The Netherlands; ^2^ Department of Pediatric Cardiology Wilhelmina Children's Hospital, University Medical Centre Utrecht The Netherlands

**Keywords:** Amplatz, coil, congenital, heart

## Abstract

**Background:**

Minimally invasive transcatheter occlusion using Amplatz canine duct occluder (ACDO) is the treatment of choice for dogs with left‐to‐right shunting patent ductus arteriosus (PDA). However, in small dogs the femoral artery diameter is often too small to accommodate the guiding catheter required for ACDO deployment.

**Objective:**

Describe the effectiveness of transarterial implantation of Amplatzer Vascular Plug 4 (AVP‐4), the only self‐expandable nitinol mesh occlusion device which can be implanted through a 4 French diagnostic catheter, in small dogs with left‐to‐right shunting PDA.

**Animals:**

Seven client‐owned dogs.

**Methods:**

Descriptive case series. Dogs with hemodynamically relevant left‐to‐right shunting PDA and a femoral artery diameter less than 2.0 mm measured preoperatively with ultrasonography were prospectively enrolled.

**Results:**

Angiography after releasing the device showed complete immediate PDA closure in 5 dogs, where the manufacturers' recommendation were strictly followed (30%‐50% device oversizing of the ductal ampulla's diameter). Trivial residual flow on angiography in the 6th dog, whose device was slightly undersized, had resolved on echocardiography within 2 hours after placement. Marked device undersizing in the 7th dog resulted in severe residual shunting, which necessitated the addition of a coil. In this dog, the AVP‐4 embolized into the pulmonary artery within 2 weeks after placement.

**Conclusions and Clinical Importance:**

Transarterial implantation of AVP‐4 is a safe, effective and technically easy procedure for PDA occlusion in small dogs and offers a valuable alternative to coil implantation. Accurate PDA measurement and device sizing is essential to prevent residual shunting, inadvertent device embolization, and protrusion of the device into the aorta.

**Abbreviations**
ACDO: Amplatz canine duct occluder
AVP: Amplatzer Vascular Plug
PDA: patent ductus arteriosus

## 1 INTRODUCTION

Patent ductus arteriosus (PDA) is 1 of the most common congenital heart diseases in dogs together with aortic and pulmonic stenosis.^1^, ^2^ If left uncorrected, most dogs with a left‐to‐right shunting PDA develop clinical signs due to congestive left‐sided heart failure, pulmonary hypertension, and arrhythmias (such as atrial fibrillation).^3^

The Amplatz canine duct occluder (ACDO, AGA Medical Corporation, Plymouth, USA, distributed by Infiniti Medical, Huddersfield, UK) is the most commonly used transcatheter device for occlusion of left‐to‐right shunting PDA in dogs. This device is specifically adapted for transarterial embolization of PDA in dogs and has the highest reported efficacy and the lowest complication rate.^4^, ^5^ However, in small dogs, the femoral artery diameter is too small to serve as a vascular access for the required guiding catheter.^4^, ^5^ Several toy breed dogs, like the Pomeranian and Chihuahua, are predisposed for PDA.^2^ Positive results on the use of a low profile ACDO prototype have been published, but the device is commercially unavailable at this time.^6^ Currently, toy and small breed dogs whose femoral artery might be too small to accommodate the guiding catheter of an ACDO undergo either surgical ligation of their PDA or coil embolization via transarterial or transvenous route.^4^, ^7^, ^8^, ^9^ Although surgical ligation is an effective technique, it is far more invasive than catheterization and major complications (such as uncontrollable hemorrhage) occur more frequently when compared with catheterization.^10^, ^11^, ^12^ The disadvantage of coils is that residual shunting occurs in 0% to 67% of the cases and embolization of the coil into the pulmonary artery in 0% to 20% of the cases, moreover, placing them is less straight forward than placing the ACDO.^4^, ^7^, ^8^, ^9^, ^13^

Though using various types of vascular plugs in the occlusion of canine PDA has been reported, their implantation requires similar large diameter guiding catheter as required for implantation of the ACDO.^14^, ^15^ The Amplatzer Vascular Plug 4 (AVP‐4, AGA Medical Corporation, Plymouth, USA, distributed by Infiniti Medical, Huddersfield, UK) is a self‐expanding nitinol mesh occlusion device, like the ACDO, which is designed for occlusion of peripheral blood vessels in humans. However, it has also been used successfully in a subgroup of children and adult human beings for occluding PDAs with a tubular morphology.^16^ Advantages of this device is that it can be delivered via a 4 French diagnostic catheter (ie, with an outer diameter of 1.35 mm), and because the device consists of 2 symmetrical lobes it can be implanted either via the arterial or via the venous side, similarly to detachable coils.

The objectives of this study were to describe the implantation procedure and the effectiveness of AVP‐4 in a series of small dogs with left‐to‐right shunting PDA.

## 2 MATERIALS AND METHODS

The study design is a descriptive case series. Descriptive statistics are reported as median and range.

### 2.1 Animals

All dogs with hemodynamically relevant left‐to‐right shunting PDA and a femoral artery diameter of less than 2.0 mm measured preoperatively with 2‐dimensional ultrasonography that were presented to the veterinary teaching hospital of Utrecht University in the Netherlands in a 1 year period (May 2019‐April 2020).

The maximal internal diameter of the femoral artery was measured with 2‐dimensional gray‐scale ultrasonography on cross‐sectional images using a 4 to 10 MHz phased array transducer with “small parts” preset (S4‐10, Logiq S8 Vet, General Electric, Boston, USA) at the inguinal area of the right hind limb, as proximal as possible.^17^ The measurement was performed on a still image on the frame where the maximum diameter of the pulsating artery was appreciated.

Information extracted from the digital medical records included age, breed, body weight, sex, femoral artery diameter on preoperative ultrasonographic images, angiographic PDA morphology, minimal and maximal ductal diameter and ductal length on the angiographic images, vascular plug diameter, vascular plug diameter/ampulla diameter ratio (measured on angiography), residual flow directly after PDA closure (visualized with angiography and in individual cases additionally with color Doppler echocardiography), and the presence of a heart murmur auscultated by the referring veterinarian 2 weeks after surgery.

The diagnosis of left‐to‐right shunting PDA was suspected in all dogs by auscultation based on the presence of a continuous murmur with the point of maximal intensity at the region of the left heart base, and it was confirmed with transthoracic 2‐dimensional, color Doppler and spectral Doppler echocardiography, by visualization of the PDA and a continuous high velocity flow arising from the region of the pulmonic bifurcation toward the pulmonic valve.^18^

### 2.2 Implantation procedure

General anesthesia was achieved using a standard protocol, which included premedication with IV administered methadone and induction with alfaxalone administered IV and maintenance anesthesia with remifentanil in constant rate infusion and isoflurane vaporized in a mixture of oxygen and air (1 : 1) administered by mechanical ventilation. Anesthesia monitoring consisted of continuous pulse oximetry, capnography, electrocardiography, core temperature (measured with a rectal or esophageal thermometer probe), and intermittent noninvasive (oscillometric) blood pressure measurement. Postoperative therapy consisted of meloxicam administered PO for 1 to 4 days at the discretion of the attending anesthesiologist. No heparin or prophylactic antibiotics were administered in the perioperative period.^19^

The dogs were placed in right lateral recumbency on the fluoroscopy table and the area of the right femoral artery was clipped and prepared for surgery. A marking catheter (Marker, Infiniti Medical, Huddersfield, UK) was placed in the esophagus. The right femoral artery was accessed via surgical cut‐down. The distal end of the femoral artery was ligated with an absorbable multifilament suture (polyglactin 910, Vicryl Plus 3‐0 Ethicon, Johnson & Johnson International, Diegem, Belgium). A 17‐gauge peripheral venous catheter (Vasofix Branüle, B. Braun, Melsungen, Germany, ref 4268156B) was placed in the femoral artery through which a 0.038 in., 150 cm guide wire (Emerald Guidewire, Standard Straight Tip, Cordis, Dublin, Ireland, ref 502‐541) was introduced. The peripheral catheter was then removed and a 4 French, 65 cm diagnostic catheter with a 0.038 in. lumen and an outer diameter of 1.35 mm (Tempo 4, multipurpose MP B2, Open End, 2 side holes, Cordis, Dublin, Ireland, ref 451‐408V2) was inserted into the femoral artery over the guidewire.

Angiography was performed by injection of approximately 1.5 mL/kg iodinated contrast agent (350 mg I/mL) with an automated injector (21 mL/s, with a maximum pound per square inch [PSI] of 1000) through the diagnostic 4 French catheter (with end‐hole and side‐holes) with its tip in the descending aorta at the level of the tracheal bifurcation. The tip of the catheter was directed in such a way that traumatic injury by the high velocity contrast jet to the vascular wall was prevented. Minimal ductal diameter, and the diameter and the length of the ductal ampulla were measured. After the angiography, invasive pressure measurements were performed in the descending aorta and in the pulmonary trunk, which is a standard procedure in the authors' catheterization laboratory.

**FIGURE 1:**
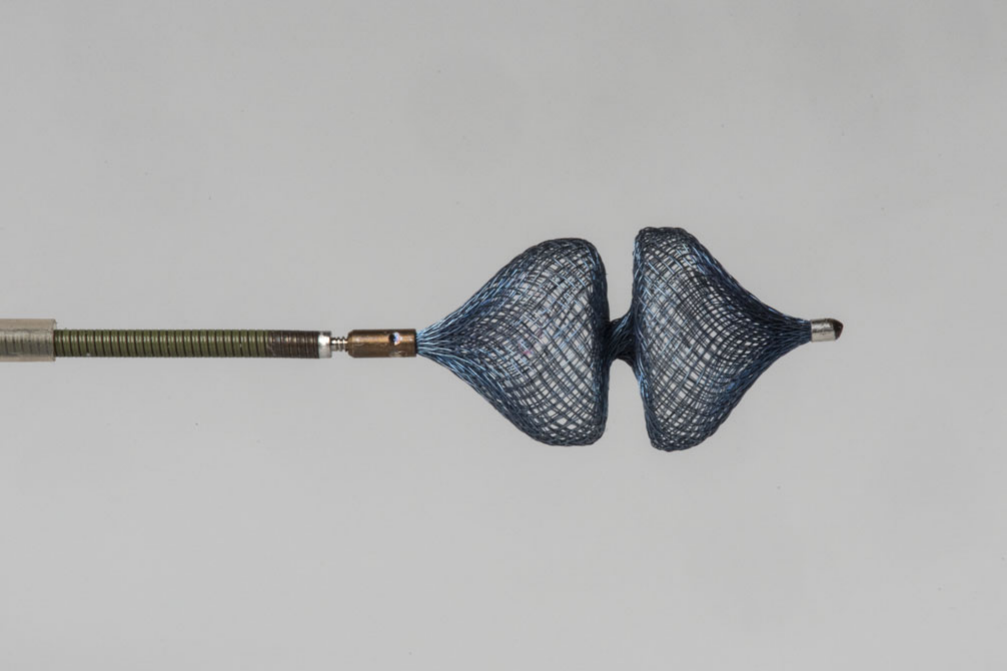
Amplatzer Vascular Plug 4. The plug consists of 2 symmetrical lobes and has a radiopaque marker band at each end

The AVP‐4 was selected based on its diameter, so as to be 1.3 to 1.5 times the maximal diameter of the ductal ampulla (ie, 30%‐50% oversizing), according to the manufacturer's recommendations for occlusion of peripheral vessels in humans. The device is available in 5 sizes based on the maximum diameter of each of the 2 symmetrical lobes, ranging from 4 to 8 mm with 1 mm increments. The length of the unconstrained (fully deployed) plugs range from 10.0 to 13.5 mm. Length is related to the plug diameter with large diameters having longer lengths (Figure 1). The delivery of the device took place through the 4 French diagnostic catheter, in a similar way to the implantation procedure of an ACDO.^20^ With the tip of the catheter in the pulmonary trunk, the 1st (distal) lobe of the device was unsheathed by holding the delivery wire in place and retracting the diagnostic catheter. Next, the delivery wire together with the diagnostic catheter were withdrawn until a resistance was felt at the pulmonary ostium. The 2nd lobe of the device was unsheathed in the ductal ampulla by further withdrawing the diagnostic catheter over the delivery wire. The length of the fully deployed device as observed on the angiogram, was subjectively compared with the length of the ductal ampulla on the initial angiogram. When the ductal ampulla was judged to be long enough to accommodate the whole device without its protrusion into the aorta, the device was repositioned. The diagnostic catheter was then advanced over the delivery wire to recapture approximately ¾ of the device, allowing the device to be retracted into the PDA. After which both lobes of the device were unsheathed within the ampulla. Next, the device was gently pushed with the delivery wire to confirm the position was stable prior to release. The device was then released from the delivery wire by counter clock‐wise rotation while watching with fluoroscopy. Because of the small‐gauge delivery catheter, which was filled with the delivery wire, it was not possible to check the position of the device with a diluted contrast injection along the delivery wire before releasing the device. A repeated angiography was performed 10 minutes after release of the device. After removing the angiographic catheter, the femoral artery was ligated proximal to the vascular opening and the surgical wound was closed in 3 layers with an absorbable monofilament suture material (Monocryl Plus 4‐0, Ethicon, Johnson & Johnson International, Diegem, Belgium). After completion of the surgery, thoracic radiographs were obtained on the angiography table while the dog was still under general anesthesia to document the position of the device. A transthoracic echocardiographic examination was performed after recovery on the same day in dogs whose PDA showed residual shunting on the final angiogram. Dogs were discharged from the clinic the same day.

## 3 RESULTS

In the study period, 7 dogs underwent a transarterial embolization with an AVP‐4 device of a left‐to‐right shunting PDA. The median age of the dogs was 4 months (range, 2‐6 months), with a median weight of 2.3 kg (range, 1.7‐5.4 kg). The following breeds were represented: French bulldog, Jack Russell terrier, Labradoodle, Pomeranian, Welsh corgi cardigan and 2 mixed breeds (a Chihuahua and Shih tzu mix, and a miniature Poodle and miniature Pinscher mix). The study sample consisted of 1 male and 6 females.

All dogs were asymptomatic at the time of surgery. All dogs had a continuous murmur with the point of maximal intensity at the region of the left heart base on physical examination. A hemodynamically relevant left‐to‐right shunting PDA was confirmed with signs of left ventricular volume overload, as varying degree of eccentric hypertrophy on transthoracic echocardiography in all dogs. The median normalized left ventricular lumen diameter in diastole was 2.01 (range, 1.71‐2.62; reference, 1.27‐1.85).^18^ No concurrent congenital cardiac defects were identified in any of the dogs.

Median femoral artery diameter was 1.6 mm (1.25‐1.80 mm) measured by 2‐dimensional gray scale ultrasonography.

The angiographic PDA morphology was classified as type IIA (n = 5), type IIB (n = 1) and in 1 dog the ostium could not be clearly identified, but the morphology classification was presumed to be type III (n = 1).^21^ The median maximal diameter of the ductal ampulla was 4.5 mm (range, 3.6‐10.0 mm). The minimal ductal diameter was localized at the level of the pulmonary ostium in all dogs and its median diameter was 2.3 mm (range, 1.6‐4.0 mm). The median length of the ductal ampulla was 11.0 mm (range, 7.7‐14.0 mm). The median plug diameter was 6 mm (5‐8 mm) and the median plug diameter/ampulla diameter ratio was 1.38 (0.80‐1.51).

**FIGURE 2:**
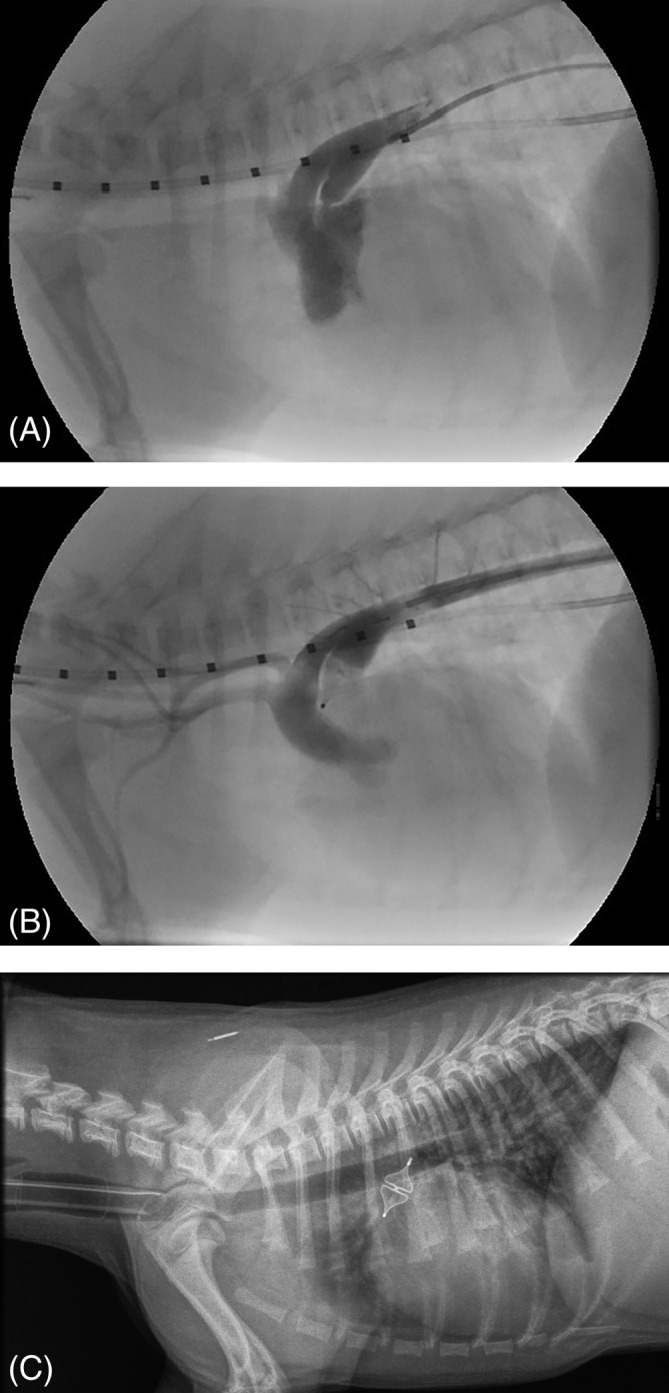
A, Angiographic image of a 4‐month‐old Jack Russell terrier with a left‐to‐right shunting patent ductus arteriosus (PDA) with the dog in right lateral recumbency. Iodinated radiographic contrast material injected in the descending aorta via a diagnostic 4 French catheter reveals a left‐to‐right shunting patent ductus arteriosus with a type IIA morphology and appearance of the contrast material in the pulmonary trunk. The marking catheter with 1 cm increments positioned in the esophagus is used for calibration. B, Angiography after implanting the Amplatzer Vascular Plug 4 (AVP‐4) in the ductal ampulla in the same dog as (A) shows a complete occlusion of the PDA. No contrast can be seen in the pulmonary trunk. C, Lateral thoracic radiograph of the same dog as shown on (A) and (B) directly after transcatheter occlusion of a left‐to‐right shunting PDA with the AVP‐4 with the dog still under general anesthesia and intubated. The symmetrical shape of the expanded device can be appreciated

The whole device was placed in the ampulla in 5 dogs (Figure 2A,B). In 2 dogs, the device was intentionally placed with the distal lobe of the device in the pulmonary trunk and the 2nd proximal lobe in the ductal ampulla. In these 2 dogs, deployment of the whole device (both lobes) within the ampulla would have resulted in device protrusion into the aorta. The device remained in position in all 7 dogs after release, and this was also documented with postoperative radiographs after completing the procedure (Figure 2C). The median duration of the procedure was 103 minutes (range, 76‐134 minutes). Placing the AVP‐4 was subjectively a technically simple procedure to perform, comparable to that of an ACDO.

**FIGURE 3:**
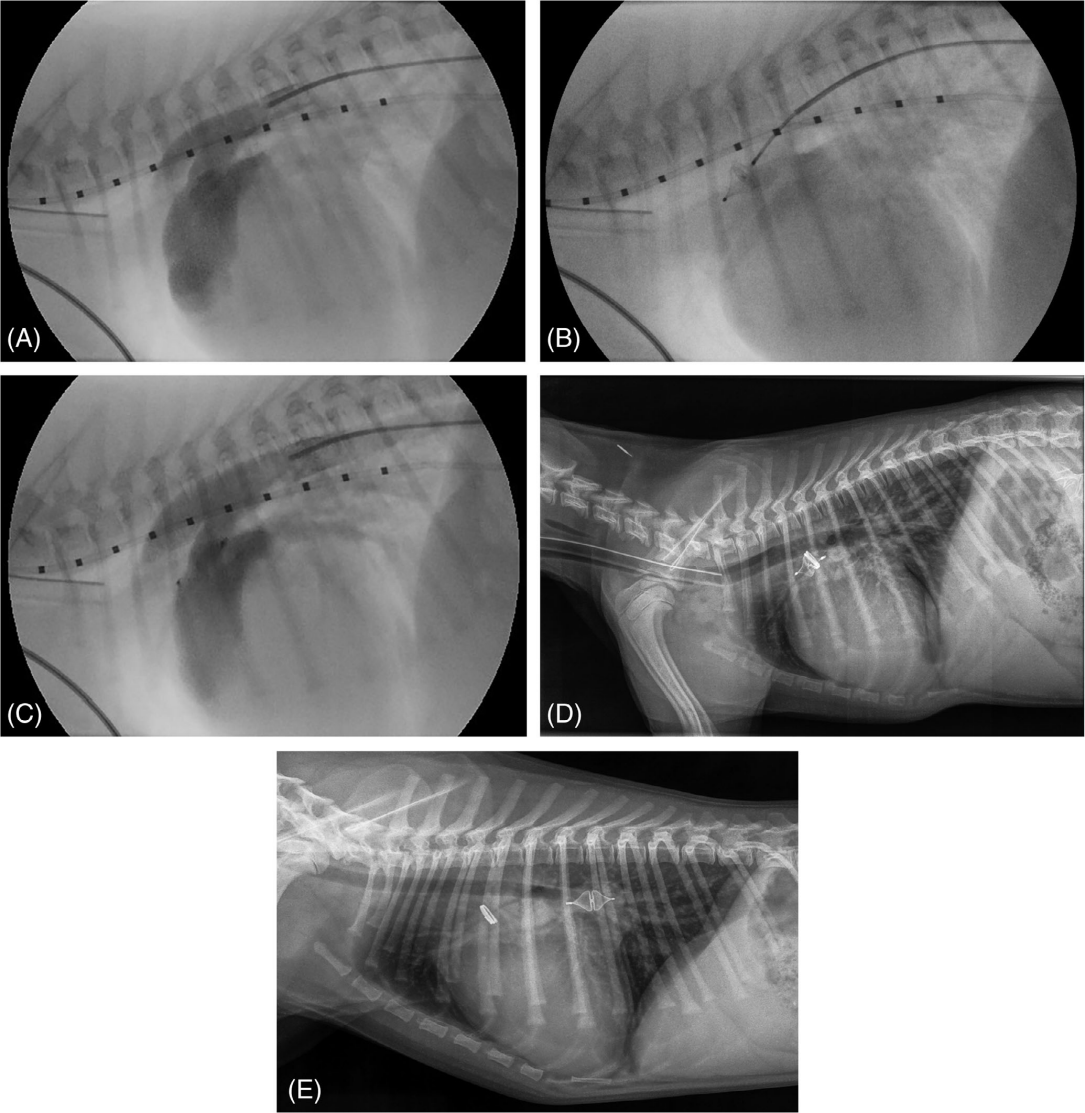
A, Angiographic image of a 2‐month‐old Labradoodle with a left‐to‐right shunting patent ductus arteriosus (PDA) with the dog in right lateral recumbency. Iodinated radiographic contrast material injected in the descending aorta via a diagnostic 4 French catheter reveals a left‐to‐right shunting patent ductus arteriosus with an assumed type III morphology and appearance of the contrast material in the pulmonary trunk. The diameter of the ostium of the PDA is difficult to assess. The marking catheter with 1 cm increments positioned in the esophagus is used for calibration. B, Fluoroscopic image of the implantation procedure of the AVP‐4 in the ductal ampulla of the same dog as shown on (A). The device is still attached to the delivery wire. Gentle pressure on the device causes the deformation of the device suggesting a stable position in the ductal ampulla. C, Angiographic image of the same dog as shown on (A) and (B) after releasing the AVP‐4 in the ductal ampulla reveals a large amount of residual shunting. The device is located still partly in the PDA. D, Lateral thoracic radiograph of the same dog whose fluoroscopic images are shown in (A)‐(C) directly after transcatheter occlusion of a left‐to‐right shunting PDA with the AVP‐4 and an additionally placed 8 mm coil in the ductal ampulla. The dog was still under general anesthesia and intubated. The position of the AVP‐4 and the coil is the same as on the intraoperative fluoroscopic images. Immediately after taking this radiograph, in an awake dog, cardiac auscultation revealed no murmur and color Doppler echocardiography showed no residual shunting. E, Lateral thoracic radiograph of the same dog whose images are shown in (A)‐(D) 2 weeks after transcatheter occlusion of a left‐to‐right shunting PDA with a AVP‐4 of 8 mm diameter and an additionally placed 8 mm coil in the ductal ampulla. The 8 mm AVP‐4 has embolized into the right pulmonic artery and the 8 mm coil is still in its original position in the ductal ampulla

No residual flow was visualized with angiography after releasing the device in 5 of the 7 dogs. In the 6th dog, a trivial residual flow was visualized. In this dog, the largest available plug (8 mm in diameter) was implanted, and the plug diameter/ampulla diameter ratio was 1.18. No residual shunting was observed by color Doppler echocardiography immediately after the procedure. The 7th dog with residual shunting showed severe amount of shunting on angiography after releasing the device. In this dog, the ostium could not be accurately identified and measured on the initial angiogram (Figure 3A). The largest (8 mm) device was chosen, but the 1st lobe of the device could fairly easily be pulled through the PDA ostium from the pulmonary trunk into the aorta. At this point, the owner was contacted by phone to explain that the largest available device was too small to safely close the PDA. The advice was to stop the procedure and to close the PDA by surgical ligation or repeat the catheterization procedure through a transvenous approach after ordering an appropriately sized different occlusion device (such as an AVP‐2). The owner did not want to consider another surgery and an agreement was reached as follows: if a stable position of the device could be obtained, the device would be released. With some manipulation, it was possible to place the device in the ampulla in such a way that it could not be pushed through the PDA into the pulmonary trunk (Figure 3B). After release of the device, it stayed in position. Because angiography after 10 minutes revealed a large amount of residual shunting (Figure 3C), an 8 mm coil (MReye Embolization Coil, Cook Medical, European Distribution, Baesweiler, Germany) was additionally placed in the ampulla to try to obtain a complete closure of the PDA. Angiography was repeated 10 minutes after releasing the coil and showed a decreased, but still a large amount of shunting. It was decided not to place any more coils and finish the procedure (Figure 3D). Immediately after the procedure, no murmur was auscultated and no shunting was observed on color Doppler echocardiography in this dog. Two weeks after the procedure a murmur with an intensity of 3 to 4 out of 6 was auscultated by the referring veterinarian when the dog came in for removing the skin sutures. A lateral thoracic radiograph was obtained and showed that the vascular plug had embolized into the right pulmonary artery and the coil was still in its original position in the ductal ampulla (Figure 3E). The plug diameter/ampulla diameter ratio in this dog was 0.80 (using the largest available plug) and the plug diameter was 2 mm smaller than the maximal diameter of the ductal ampulla. The ostium diameter could not be clearly identified but was estimated to be 4 mm, which was apparently wrong as the 8 mm device could be pulled through the ostium. Retrieval of the plug and PDA closure were not performed in a 2nd procedure due to lack of owner consent. The owner has not noticed any problems with the puppy after the surgery and the dog is reported to be clinically healthy 8 months after the procedure.

**FIGURE 4:**
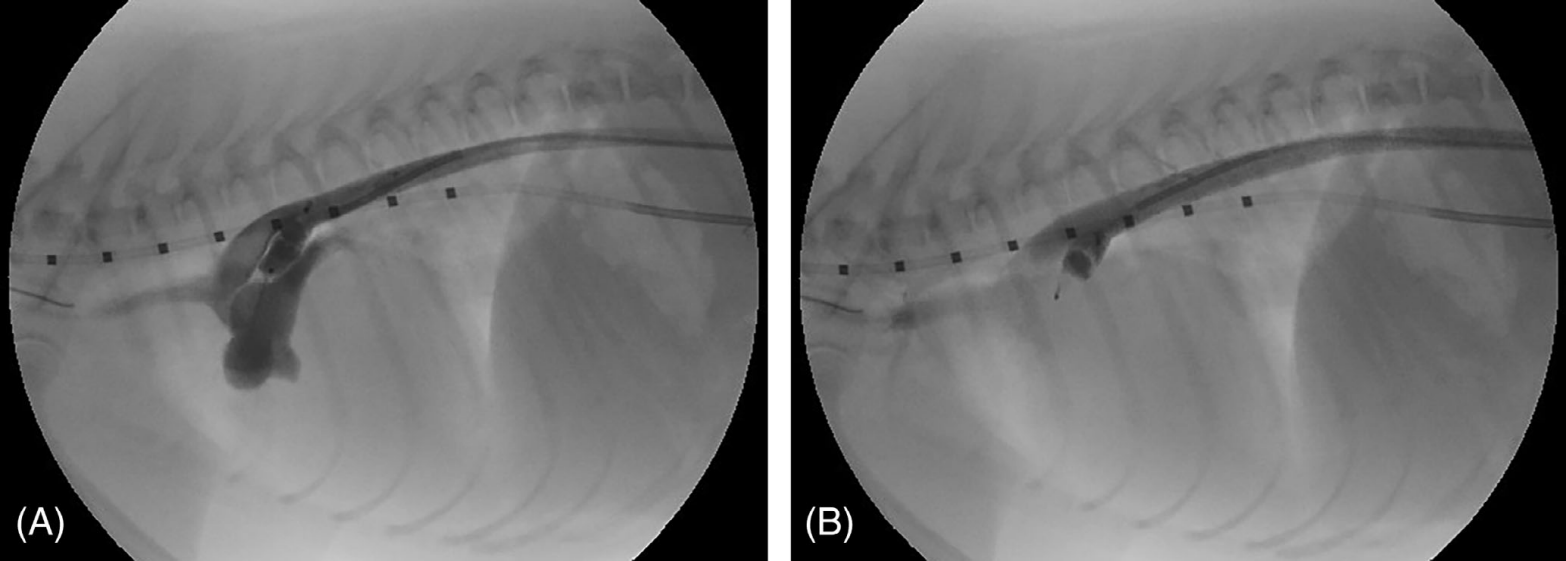
A, Angiographic image of a 6‐month‐old Pomeranian with a left‐to‐right shunting patent ductus arteriosus (PDA) with the dog in right lateral recumbency 10 minutes after releasing the AVP‐4 in the ductal ampulla. The most dorsally located tip of the device protrudes into the aorta and there is severe residual shunting present, as the pulmonary trunk contains a large amount of contrast. The most ventrally located tip of the device is situated in the ductal ampulla. B, Angiographic image of the same dog as shown on (A), 20 minutes after releasing the AVP‐4 in the ductal ampulla, and 10 minutes after the aortogram shown in (A). Residual shunting is no longer observed as no contrast can be seen in the pulmonary trunk. The device has moved, without any manipulation toward the PDA ostium. As a result, the device is no longer protruding into the aorta, and its ventrally located tip is probably situated in the pulmonary trunk through the pulmonary ostium of the PDA, which is presumably responsible for the complete occlusion of the PDA

In 1 dog, a spontaneous device movement was observed between 10 and 20 minutes after device release. Angiography 10 minutes after release showed that the device protruded into the aorta and the aortogram showed severe residual shunting (Figure 4A). However, 10 minutes later (20 minutes after device release) the residual shunting was no longer observed, whereas the device moved by itself deeper into the ampulla toward the pulmonary trunk and therefore it was no longer protruding into the aorta and obtained complete ductal occlusion (Figure 4B).

Continuous heart murmur resolved in all dogs on the day of the surgery. Two weeks after the procedure, all dogs went to the referring veterinarian for removing the skin sutures. All dogs were asymptomatic. Information on heart auscultation at that time was available for all 7 dogs. In 1 of them a heart murmur was auscultated, this was the dog in which embolization of the device occurred. In the remaining 6 dogs, no heart murmur was auscultated. No complications were observed during or directly after the procedure in any of the dogs.

## 4 DISCUSSION

The present study shows that transarterial occlusion of PDA in small dogs with the AVP‐4 is a feasible and effective technique. Complete immediate occlusion of the PDA was achieved in all dogs where the sizing was performed according the manufacturers' recommendation for occlusion of peripheral vessels in humans (30%‐50% device oversizing of the ductal ampulla's diameter).

In 1 dog, in which trivial residual flow was visualized with angiography the plug was slightly undersized, with a plug diameter/ampulla diameter ratio of 1.18. In this dog, no residual shunting was observed by color Doppler echocardiography after surgery. However, in another dog in this study both residual shunting and embolization of the plug within 2 weeks after surgery could be explained by the use of a markedly undersized plug (device diameter/ampulla diameter ratio of 0.80 instead of the recommended 1.3‐1.5). In a study on children with tubular PDA, the selection of the device was chosen to be at least 1 mm larger than the diameter of the largest portion of the PDA.^16^ In the present study, the dog in which the plug embolized was the only dog in which the device diameter was not 1 mm larger than the diameter of the largest portion of the PDA.

Device embolization into the pulmonary artery is described with the use of the ACDO. Embolization just after releasing the device during the procedure is described in 2 dogs, in which successful retrieval of the device was accomplished.^22^ In another case report, delayed embolization 2 days after discharge is described and this dog died the same day.^23^

It remains difficult to explain why in the dog of the present study, the coil with the same diameter as the embolized AVP‐4 did stay in its original position in the ductal ampulla. Also, according to the owner, the embolized plug did not result in apparent clinical signs.

The unconstrained length of the AVP‐4 varies between 10.0 and 13.5 mm. The ratio between the plug diameter and the ductal ampulla diameter will determine the amount of constraint of the plug and therefore possible elongation of the device beyond the reported unconstrained length. The median ampulla length in this study was 11.0 mm (range, 7.7‐14.0 mm). If this ductal length is compared to the unconstrained length of the plug, it is likely that in some dogs the length of the partially constrained plug could potentially limit its use. In these cases, (part of) 1 of the lobes of the device will protrude in either the pulmonary trunk or the aorta depending on the location of release. Letting the device protrude into the aorta could potentially lead to localized narrowing of the aorta. This can be prevented by adequate ductal length measurement and device selection. In case the selected device is longer than the ductal ampulla, there are several options. One option is to place the distal lobe of the device in the pulmonary artery. Another option is to select a smaller diameter (and therefore shorter length) AVP‐4, as long as the plug diameter remains within the 30% to 50% oversizing range. Lastly, instead of an AVP‐4 a short detachable coil can be selected. Unlike the ACDO, the distal lobe of the AVP‐4 that would protrude in the pulmonary artery is not flat. Leaving the distal lobe of the device in the pulmonary artery could potentially lead to (left) pulmonary artery stenosis and secondary pressure overload of the right ventricle. This scenario is probably most likely in small dogs, when the occlusion is done after about 9 months of age, when the dog is not expected to grow postoperatively. The presence and severity of left pulmonary artery stenosis could be evaluated by auscultation and Doppler echocardiography after PDA occlusion. In the current study, Doppler studies were not performed after the procedure in the 2 dogs in which a part of the device was intentionally placed in the pulmonary artery, because neither of them had a murmur. In none of our dogs, the device was protruding into the aorta based on the final angiogram. Device‐related left pulmonary artery stenosis can improve over time in children with growth and without complications.^24^ To our knowledge, there is no study published on device‐related pulmonary artery stenosis in dogs.

Assessing ductal length on transthoracic echocardiography before surgery is difficult and therefore does not aid in selecting the appropriate technique for PDA closure.^25^ Transesophageal echocardiography (TEE) could be of benefit as an additional or sole imaging technique to assess ductal dimensions during the occlusion procedure.^26^, ^27^, ^28^, ^29^, ^30^ In small dogs, the size (diameter) of the TEE probe can be a limiting factor, even if a pediatric probe is used. Unfortunately, TEE is not available in our clinic. It would have been useful in 1 of the dogs in our study, in which the pulmonary ostium could not be clearly identified on an angiogram.

Occlusion of PDA in small dogs can also be achieved with surgical ligation or transcatheter coil embolization.^7^, ^8^, ^9^, ^13^ Surgical ligation has a high success rate and low complication rate with experienced surgeons, but when hemorrhage occurs perioperative death rate is high.^10^, ^11^, ^12^ Implantation of coils can be performed by a transvenous or transarterial approach and by an uncontrolled release system or detachable coils.^7^, ^8^, ^9^ The downside of implantation of coils is that both residual shunting and embolization occur relatively frequent (0%‐67% and 0%‐20%, respectively).^4^, ^7^, ^8^, ^9^ Another complication that can occur with coil implantation is intravascular hemolysis, this is not reported in dogs with the use of self‐expandable nitinol mesh closure devices.^8^, ^31^, ^32^ An advantage of AVP‐4 compared to coils is that the ratio between the ductal ostium and ductal ampulla is less important than in case of coil implantation. Because the size of the coil is selected based on the ostium diameter, in cases when the ostium and the ampulla diameters differ only slightly, the coil might take an oblique position in the ductal ampulla resulting in an incomplete occlusion.

In small dogs the transvenous approach is often used to deliver a detachable coil. The advantage of the transvenous approach is that the (femoral or jugular) veins can accommodate a larger catheter than the femoral artery and there is lower risk of bleeding from the vein than from the artery if percutaneous puncture is used instead of a surgical cut down.^4^, ^7^, ^8^ The challenge of the transvenous approach is that it is more difficult to enter the ductus from the pulmonary artery side and therefore the fluoroscopy time is longer.^4^ There is also a higher risk of arrhythmias due to passing the right ventricle with a catheter.^13^ Although in our study no transvenous procedure was performed, the symmetrical structure of the AVP‐4 makes a transvenous procedure also possible.

Possible complications that can occur with the use of vascular plugs in general are embolization, recanalization, hemolysis, air embolism, thrombosis, ductal tear, infection, and allergic reactions. Previous studies on the AVP‐1 in dogs revealed pulmonic embolization, transient hindlimb lameness, bruising/pruritus around the surgical wound, rotation of the device resulting in a marked increase in flow, delayed recanalization or movement and lack of thrombosis due to von Willebrand disease.^4^, ^14^, ^15^

The reason the AVP‐4 was selected in the present study instead of other members of the Amplatzer vascular plug family is that the AVP‐4 (all available sizes) is the only plug that can be delivered through a 4 French (outer diameter) diagnostic catheter and its elongated tapered design makes it fit perfectly into the PDA type II (most common type of) canine ductal ampulla. The AVP‐1‐3 all need at least a 4 French (inner diameter) sheath, which size can be a limitation in small dogs. Insufficient thrombus formation is less likely with the newer AVP versions (AVP‐2‐4) compared to the AVP‐1 because the manufacturer increased the number of layers and lobes/segments.^33^ The 3 segments of the AVP‐2 and the 2 segments of the AVP‐3 and AVP‐4 increase the surface area of the contact point, theoretically making migration of the device less likely compared to the AVP‐1, which consists of 1 segment. A possible disadvantage of AVP‐4 is its relative long uncompressed length (10.0‐13.5 mm) compared to the AVP‐1 (7‐8 mm), AVP‐2 (6‐18 mm) and AVP‐3 (6.5 mm), which might result in protrusion of the plug into the aorta if a PDA is short.^33^, ^34^

Using AVP‐2 for ductal closure in small dogs with a large PDA could be an option, because it is available in larger diameters than the AVP‐4. The symmetrical design of the AVP‐2 makes it possible to implant it via the arterial or via the venous side. The major disadvantage of AVP‐2 is that it requires a larger lumen delivery catheter than 4 French. However, if the femoral artery is too small for implanting an ACDO, and the diameter of the PDA (ampulla >6 mm) requires a larger plug than the largest available size AVP‐4 (8 mm), an AVP‐2 could probably be a good option, but it needs to be implanted in a retrograde fashion through the femoral or jugular vein.

Intraoperative invasive pressure measurements are part of the standard procedure of transarterial PDA closure at the authors' institution when a PDA is occluded with any type of implant. The reasons for this are to make sure that the tip of the catheter is located in the pulmonary artery before the implant is being delivered, to assess the pulmonary pressure (presence and severity of pulmonary hypertension) and to assess the immediate effect of ductal occlusion on the systemic arterial blood pressure. In addition, in cases where an implant from the PDA protrudes into the aortic lumen, particularly in case of AVP‐4, invasive pressure measurements in the aorta cranial and caudal to the implant can reveal whether there is a relevant pressure gradient present, which would necessitate additional steps, such as removing the implant or trying to adjust its position.

Limitations of the present study are the small number of dogs included and the lack of long term follow up. In addition, in all reported dogs only follow up information on physical examination from the referring veterinarian was available. Also, comparison between the different occlusion techniques was not performed, but this was not attempted in this study. Therefore, it is not possible to objectively conclude which technique for PDA occlusion in small dogs is superior.

## CONFLICT OF INTEREST DECLARATION

Authors declare no conflict of interest.

## OFF‐LABEL ANTIMICROBIAL DECLARATION

Authors declare no off‐label use of antimicrobials.

## INSTITUTIONAL ANIMAL CARE AND USE COMMITTEE (IACUC) OR OTHER APPROVAL DECLARATION

Authors declare no IACUC or other approval was needed.

## HUMAN ETHICS APPROVAL DECLARATION

Authors declare human ethics approval was not needed for this study.

## References

[1] Schrope DP . Prevalence of congenital heart disease in 76,301 mixed‐breed dogs and 57,025 mixed‐breed cats. J Vet Cardiol. 2015;17:192‐202. 26363941 10.1016/j.jvc.2015.06.001

[2] Oliveira P , Domenech O , Silva J , Vannini S , Bussadori R , Bussadori C . Retrospective review of congenital heart disease in 976 dogs. J Vet Intern Med. 2011;25:477‐483. 21418326 10.1111/j.1939-1676.2011.0711.x

[3] Eyster GE , Eyster JT , Cords GB , Johnston J . Patent ductus arteriosus in the dog: characteristics of occurrence and results of surgery in one hundred consecutive cases. J Am Vet Med Assoc. 1976;168:435‐438. 1254517

[4] Singh MK , Kittleson MD , Kass PH , Griffiths LG . Occlusion devices and approaches in canine patent ductus arteriosus: comparison of outcomes. J Vet Intern Med. 2012;26:85‐92. 22211471 10.1111/j.1939-1676.2011.00859.x

[5] Gordon SG , Saunders AB , Achen SE , et al. Transarterial ductal occlusion using the Amplatz® canine duct occluder in 40 dogs. J Vet Cardiol. 2010;12:85‐92. 20615776 10.1016/j.jvc.2010.04.004

[6] Stauthammer CD , Olson J , Leeder D , Hohnadel K , Hanson M , Tobias AH . Patent ductus arteriosus occlusion in small dogs utilizing a low profile Amplatz® canine duct occluder prototype. J Vet Cardiol. 2015;17:203‐209. 26363940 10.1016/j.jvc.2015.06.002

[7] Henrich E , Hildebrandt N , Schneider C , Hassdenteufel E , Schneider M . Transvenous coil embolization of patent ductus arteriosus in small (≤3.0 kg) dogs. J Vet Intern Med. 2011;25:65‐70. 21092005 10.1111/j.1939-1676.2010.0637.x

[8] Blossom JE , Bright JM , Griffiths LG . Transvenous occlusion of patent ductus arteriosus in 56 consecutive dogs. J Vet Cardiol. 2010;12:75‐84. 20594928 10.1016/j.jvc.2010.04.002

[9] Hogan DF , III HWG , Gordon S , Miller MW . Transarterial coil embolization of patent ductus arteriosus in small dogs with 0.025‐inch vascular occlusion coils: 10 cases. J Vet Intern Med. 2004;18:325‐329. 15188819 10.1892/0891-6640(2004)18<325:tceopd>2.0.co;2

[10] Hunt GB , Simpson DJ , Beck JA , et al. Intraoperative hemorrhage during patent ductus arteriosus ligation in dogs. Vet Surg. 2001;30:58‐63. 11172461 10.1053/jvet.2001.20339

[11] Goodrich KR , Kyles AE , Kass PH , Campbell F . Retrospective comparison of surgical ligation and transarterial catheter occlusion for treatment of patent ductus arteriosus in two hundred and four dogs (1993‐2003). Vet Surg. 2007;36:43‐49. 17214819 10.1111/j.1532-950X.2007.00233.x

[12] Ranganathan B , LeBlanc NL , Scollan KF , et al. Comparison of major complication and survival rates between surgical ligation and use of a canine ductal occluder device for treatment of dogs with left‐to‐right shunting patent ductus arteriosus. J Am Vet Med Assoc. 2018;253:1046‐1052. 30272512 10.2460/javma.253.8.1046

[13] Schneider M , Hildebrandt N , Schweigl T , Schneider I , Hagel KH , Neu H . Transvenous embolization of small patent ductus arteriosus with single detachable coils in dogs. J Vet Intern Med. 2001;15:222‐228. 11380031 10.1892/0891-6640(2001)015<0222:teospd>2.3.co;2

[14] Achen SE , Miller MW , Gordon SG , Saunders AB , Roland RM , Drourr LT . Transarterial ductal occlusion with the amplatzer vascular plug in 31 dogs. J Vet Intern Med. 2008;22:1348‐1352. 18798793 10.1111/j.1939-1676.2008.0185.x

[15] Smith PJ , Martin M . Transcatheter embolisation of patent ductus arteriosus using an amplatzer vascular plug in six dogs. J Small Anim Pract. 2007;48:80‐86. 17286660 10.1111/j.1748-5827.2006.00255.x

[16] Baruteau A , Lambert V , Riou J , et al. Closure of tubular patent ductus arteriosus with the Amplatzer Vascular Plug IV: feasibility and safety. World J Pediatr Congenit Heart Surg. 2015;6:39‐45. 25548342 10.1177/2150135114558070

[17] Szatmári V . Chitosan hemostatic dressing for control of hemorrhage from femoral arterial puncture site in dogs. J Vet Sci. 2015;16:517‐523. 26119165 10.4142/jvs.2015.16.4.517 PMC4701745

[18] Boon JA . Veterinary Echocardiography. Ames, Iowa, USA: Wiley/Blackwell; 2011.

[19] Szatmári V . Incidence of postoperative implant‐related bacterial endocarditis in dogs that underwent trans‐catheter embolization of a patent ductus arteriosus without intra‐ and post‐procedural prophylactic antibiotics. Vet Microbiol. 2017;207:25‐28. 28757032 10.1016/j.vetmic.2017.05.023

[20] Nguyenba TP , Tobias AH . The Amplatz® canine duct occluder: a novel device for patent ductus arteriosus occlusion. J Vet Cardiol. 2007;9:109‐117. 18054306 10.1016/j.jvc.2007.09.002

[21] Miller MW , Gordon SG , Saunders AB , et al. Angiographic classification of patent ductus arteriosus morphology in the dog. J Vet Cardiol. 2006;8:109‐114. 19083344 10.1016/j.jvc.2006.07.001

[22] Lavennes M , Chetboul V , Passavin P , et al. Successful transcatheter retrieval of embolized Amplatz canine duct occluders in two dogs. J Vet Cardiol. 2018;20:451‐457. 30217498 10.1016/j.jvc.2018.07.010

[23] Carlson JA , Achen SA , Saunders AB , Gordon SG , Miller MW . Delayed embolization of an Amplatz® canine duct occluder in a dog. J Vet Cardiol. 2013;15:271‐276. 24246437 10.1016/j.jvc.2013.10.001

[24] Tomasulo CE , Gillespie MJ , Munson D , et al. Incidence and fate of device‐related left pulmonary artery stenosis and aortic coarctation in small infants undergoing transcatheter patent ductus arteriosus closure. Catheter Cardiovasc Interv. 2020;96:889‐897. 32339400 10.1002/ccd.28942

[25] Schneider M , Hildebrandt N , Schweigl T , Wehner M . Transthoracic echocardiographic measurement of patent ductus arteriosus in dogs. J Vet Intern Med. 2007;21:251‐257. 17427385 10.1892/0891-6640(2007)21[251:temopd]2.0.co;2

[26] Silva J , Domenech O , Mavropoulou A , Oliveira P , Locatelli C , Bussadori C . Transesophageal echocardiography guided patent ductus arteriosus occlusion with a duct occluder. J Vet Intern Med. 2013;27:1463‐1470. 24118205 10.1111/jvim.12201

[27] Saunders AB , Miller MW , Gordon SG , Bahr A . Echocardiographic and angiographic comparison of ductal dimensions in dogs with patent ductus arteriosus. J Vet Intern Med. 2007;21:68‐75. 17338152 10.1892/0891-6640(2007)21[68:eaacod]2.0.co;2

[28] Doocy KR , Nelson DA , Saunders AB . Real‐time 3D transesophageal echocardiography‐guided closure of a complicated patent ductus arteriosus in a dog. J Vet Cardiol. 2017;19:287‐292. 28571753 10.1016/j.jvc.2017.04.001

[29] Saunders AB , Achen SE , Gordon SG , Miller MW . Utility of transesophageal echocardiography for transcatheter occlusion of patent ductus arteriosus in dogs: influence on the decision‐making process. J Vet Intern Med. 2010;24:1407‐1413. 20738766 10.1111/j.1939-1676.2010.0587.x

[30] Porciello F , Caivano D , Giorgi ME , et al. Transesophageal echocardiography as the sole guidance for occlusion of patent ductus arteriosus using a canine ductal occluder in dogs. J Vet Intern Med. 2014;28:1504‐1512. 25041218 10.1111/jvim.12401 PMC4895578

[31] Glaus TM , Martin M , Boller M , et al. Catheter closure of patent ductus arteriosus in dogs: variation in ductal size requires different techniques. J Vet Cardiol. 2003;5:7‐12. 10.1016/S1760-2734(06)70039-X 19081352

[32] Israël NV , French AT , Wotton PR , Wilson N . Hemolysis associated with patent ductus arteriosus coil embolization in a dog. J Vet Intern Med. 2001;15:153‐156. 11300599 10.1892/0891-6640(2001)015<0153:hawpda>2.3.co;2

[33] Lopera J . The Amplatzer vascular plug: review of evolution and current applications. Semin Interv Radiol. 2015;32:356‐369. 10.1055/s-0035-1564810 PMC4640916 26622098

[34] Wang W , Li H , Tam M , et al. The Amplatzer vascular plug: a review of the device and its clinical applications. Cardiovasc Intervent Radiol. 2012;35:725‐740. 22526108 10.1007/s00270-012-0387-z

